# In vitro study of cartilage tissue engineering using human adipose-derived stem cells induced by platelet-rich plasma and cultured on silk fibroin scaffold

**DOI:** 10.1186/s13287-019-1443-2

**Published:** 2019-12-04

**Authors:** Imam Rosadi, Karina Karina, Iis Rosliana, Siti Sobariah, Irsyah Afini, Tias Widyastuti, Anggraini Barlian

**Affiliations:** 1 School of Life Sciences and Technology, Institut Teknologi Bandung, Bandung, West Java Indonesia; 2 HayandraLab, Yayasan Hayandra Peduli, Jakarta, DKI Jakarta Indonesia; 3 Klinik Hayandra, Yayasan Hayandra Peduli, Jakarta, DKI Jakarta Indonesia; 4 Biomedic, Universitas Indonesia, Jakarta, DKI Jakarta Indonesia

**Keywords:** Chondrogenesis of ADSCs, Salt-leached scaffold, Type 2 collagen, Type 1 collagen, Aggrecan

## Abstract

**Background:**

Cartilage tissue engineering is a promising technique for repairing cartilage defect. Due to the limitation of cell number and proliferation, mesenchymal stem cells (MSCs) have been developed as a substitute to chondrocytes as a cartilage cell-source. This study aimed to develop cartilage tissue from human adipose-derived stem cells (ADSCs) cultured on a *Bombyx mori* silk fibroin scaffold and supplemented with 10% platelet-rich plasma (PRP).

**Methods:**

Human ADSCs and PRP were characterized. A silk fibroin scaffold with 500 μm pore size was fabricated through salt leaching. ADSCs were then cultured on the scaffold (ADSC-SS) and supplemented with 10% PRP for 21 days to examine cell proliferation, chondrogenesis, osteogenesis, and surface marker expression. The messenger ribonucleic acid (mRNA) expression of type 2 collagen, aggrecan, and type 1 collagen was analysed. The presence of type 2 collagen confirming chondrogenesis was validated using immunocytochemistry. The negative and positive controls were ADSC-SS supplemented with 10% foetal bovine serum (FBS) and ADSC-SS supplemented with commercial chondrogenesis medium, respectively.

**Results:**

Cells isolated from adipose tissue were characterized as ADSCs. Proliferation of the ADSC-SS PRP was significantly increased (*p* < 0.05) compared to that of controls. Chondrogenesis was observed in ADSC-SS PRP and was confirmed through the increase in glycosaminoglycans (GAG) and transforming growth factor-β1 (TGF-β1) secretion, the absence of mineral deposition, and increased surface marker proteins on chondrogenic progenitors. The mRNA expression of type 2 collagen in ADSC-SS PRP was significantly increased (*p* < 0.05) compared to that in the negative control on days 7 and 21; however, aggrecan was significantly increased on day 14 compared to the controls. ADSC-SS PRP showed stable mRNA expression of type 1 collagen up to 14 days and it was significantly decreased on day 21. Confocal analysis showed the presence of type 2 collagen in the ADSC-SS PRP and positive control groups, with high distribution outside the cells forming the extracellular matrix (ECM) on day 21.

**Conclusion:**

Our study showed that ADSC-SS with supplemented 10% PRP medium can effectively support chondrogenesis of ADSCs in vitro and promising for further development as an alternative for cartilage tissue engineering in vivo.

## Background

Cartilage tissue engineering is considered as a solution for repairing the cartilage defect such as microtia and ostheoarthritis. Cartilage tissue engineering involves use of cells, bioactive factors, and scaffolds. Ideally, chondrocytes are the cell source for cartilage. However, due to their limited number and low proliferation, mesenchymal stem cells (MSCs) have been developed as a substitute to chondrocytes as a cartilage cell source [1]. Adipose-derived stem cells (ADSCs) are multipotent MSCs that can be differentiated into several cell types including chondrocytes [2]. Several studies reported that foetal bovine serum (FBS) supplemented with bioactive factors including transforming growth factor-beta (TGF-β) induces chondrogenesis of ADSCs [3, 4]. However, FBS is less preferred in cartilage tissue engineering because of its animal origin and the possibility of contaminants, such as prions and inflammatory reactions [5]. Human platelet-rich plasma (PRP) is also used as a substitute for FBS to increase cell proliferation [6, 7]. PRP contains more than 1100 proteins [8] and has various growth and differentiation factors, including TGF-β1, which regulates chondrogenesis [6, 7, 9]. Thus, in addition to replacing FBS as a proliferative agent, PRP can also induce chondrogenesis of ADSCs [8].

The other component of tissue engineering is the scaffold. Scaffolds act as a skeletal structure for cell attachment [10]. Silk fibroin is a candidate scaffold for cartilage tissue engineering based on its strength, porosity, biodegradability, biocompatibility, and support for cell proliferation and differentiation [10–12]. Silk fibroin scaffolds can be fabricated into 3D structures with various pore sizes and pore connectivity to regulate nutrient transport [11]. Our previous study showed that the 500 μm pore size was the best for chondrogenesis using ADSCs on silk fibroin scaffolds compared to the 300 μm and 100 μm pore sizes [13]. During chondrogenesis, ADSCs cultured on silk fibroin scaffolds synthesized glycosaminoglycans (GAG), type 2 collagen, and aggrecan, as chondrocyte biomarkers [10, 11].

The GAG are classified as chondroitin sulphates (CS), dermatan sulphates (DS), keratan sulphates (KS), and heparan sulphates (HS). KS and CS bind to aggrecan and interact with type 2 collagen in chondrocytes [14]. Furthermore, as osteogenesis usually proceeds through chondrogenesis, both processes need to be observed in a study to confirm the differentiation process that occurs. Type 1 collagen expression and mineralization are signs of osteogenic differentiation. However, chondrogenesis and osteogenesis show a negative feedback that makes it important to confirm and evaluate osteogenesis-related genes in studies on chondrogenic induction in cartilage tissue engineering [15]. This study aims to investigate the chondrogenesis of ADSCs cultured on silk fibroin scaffolds with a 500 μm pore size in PRP-supplemented medium.

## Methods

### Silk fibroin fabrication

Silk fibroin scaffolds were fabricated as described previously [13, 16]. Silk fibroin obtained from *Bombyx mori* cocoons was reconstructed into scaffolds by a salt leaching method. Degumming of silk fibroin was performed by immersion in 0.05% Na_2_CO_3_ solution. Silk fibroin was then diluted with 8 wt% CaCl_2_ formic acid solution and reconstructed into scaffolds using NaCl with ~ 500 μm particle size to form 500 μm pores. The mixture was then immersed in 70% alcohol and washed in distilled water for 3 days to remove the salt residues. The silk fibroin scaffolds were cut into 5 mm × 5 mm pieces with 1 mm thickness and were sterilized in an autoclave for 15 min at 121 °C.

### Isolation, culture, and expansion of ADSCs

The stromal vascular fraction (SVF) was isolated from lipoaspirates of four healthy patients using an enzymatic method which was H-Remedy enzyme patented by Yayasan Hayandra Peduli (patent number registration IDP000055609). Lipoaspirates digested by H-Remedy enzyme which incubated for 1 h at 37 °C, 300 rpm. After incubation, to inactivate the enzyme, the digested lipoaspirates were added low-glucose (1 g/L) Dulbecco’s modified Eagle’s medium (DMEM) containing 4 mM L-glutamine (Gibco, USA) followed by centrifugation for 5 min at 600×g. Then, the supernatant was discarded. The pellet SVF was diluted in saline solution. The cell number and viability was counted by trypan blue staining which were calculated per 10 mL of adipose tissue digested.

Isolated cells were cultured in basic growth medium containing low glucose (1 g/L) DMEM with L-glutamine (4 mM) (Gibco, USA), 10% FBS (Gibco, USA), and 1× antibiotic-antimycotic (Gibco, USA) and were incubated at 37 °C, 5% CO_2_. Medium was changed every 2–3 days. After reaching 80–90% confluency, the cells were sub-cultured and expanded to passages 2, 3, and 4 to be used for further assays.

### Characterization of ADSCs

#### Multipotency assay

ADSCs passage 2 were cultured in a 24-well plate (1 × 10^4^ cells/well) in basic growth medium. Medium was changed every 2–3 days. After cells reached 80% confluency, medium was replaced with StemPro differentiation kit (Gibco, USA) for chondrogenic, osteogenic, and adipogenic for 7 days. The cells were fixed in 10% formalin and then stained with Oil red O, alcian blue, and alizarin red for adipocytes, chondrocytes, and osteocytes, respectively. Cell differentiation was observed using an inverted microscope (OPTICA microscope, Italy).

#### Surface marker protein analysis

Cell surface marker analysis was performed by flow cytometry (Miltenyi Biotec) to confirm the stem cell characteristics of ADSCs. The cell surface markers used were CD73 allophycocyanin (APC), CD90 fluorescein isothiocyanate (FITC), and CD105 peridinin-chlorophyll-protein (PerCP) Cy5.5 as positive MSCs markers, and lineage negative marker-PE including CD34, CD45, CD11b, CD19, and human leukocyte antigen (HLA)-DR (Becton Dickinson) as positive haematopoietic cells markers. The cells (1 × 10^5^, passage 3) were stained with fluorescence-labelled probes specific to cell surface molecule. Data were obtained from 10,000 events per analysis.

### Characterization of PRP

PRP which was liquid form was obtained from Indonesian Red Cross Society (IFRC), Jl. Kramat Raya, No. 47, Central Jakarta, DKI Jakarta (10450). The time between blood drawing, PRP processing, activation, and delivery have been conducted in a day. The PRP was stored at −21 °C without light exposure.

#### Platelet, erythrocyte, and leucocyte measurement

About 200 μl of PRP was aliquot into 1.5-ml sterile microtubes. The sample was analysed using the Sysmex KX-21 automated haematology analyser, which was calibrated before analysing the blood cells and platelet counts in PRP. Platelet measurement was done twice for each PRP batch.

#### Level of TGF-β1

The protein level of TGF-β1 was determined using the enzyme-linked immunosorbent assay (ELISA) following the protocol provided by R&D systems, USA. According to manufacturer’s instruction, the standard stock solution of TGF-β1 was serially diluted to form standards of 2000 pg/ml, 1000 pg/ml, 500 pg/ml, 250 pg/ml, 125 pg/ml, 62.5 pg/ml, and 31.3 pg/ml. Next, 50 μl diluent solution RD1-73 was added to each 96 well-plate. The standard samples and solutions were then added to the 96 well-plate each and incubated for 2 h at 20−25 °C. In the next step, the wells were flushed with 400 μl wash buffer, and 100 μl TGF-β1 antibody solution was added and incubated at room temperature for 30 min in the dark. In the last step, 100 μl stop solution was added. The TGF-β1 concentration value was determined as absorbance using a microplate reader (iMark BioRad) at a wavelength of 450 nm.

#### Total protein of PRP

The total protein content of PRP was analysed using the Pierce™ Bicinchoninic acid (BCA) Protein Assay Kit (Thermo Scientific, USA). According to manufacturer’s instruction, the total protein measurement was initiated by preparing standard concentrations of 0–2000 μg/ml albumin (Thermo Scientific, USA). The samples, standards, and 50 μl control (distilled water) were then dissolved in 1 ml working reagent. Next, the samples, standards, and control were incubated at 37 °C for 30 min, followed by a room temperature (~ 20-25 °C) incubation for 5 min. The result obtained was then read at a wavelength of 562 nm using a Nanodrop One, Thermo Fisher.

### Cells proliferation assay

ADSCs at passage 4 (1 × 10^5^) were cultured on silk fibroin scaffolds (ADSC-SS) in various medium (StemPro chondrogenesis differentiation kit as positive control, 10% FBS in DMEM as negative control, and 10% PRP in DMEM). Cell growth was analysed to determine the biocompatibility of silk fibroin scaffold with ADSCs. The culture medium in the treatment and control groups of the scaffold was removed and the cells were washed with Hank’s balanced salt solution (HBSS) (Gibco, USA). On days 1, 3, 5, 7, 14, and 21, 100 μl DMEM (Gibco, USA) and the reagent of 10 μl 3-(4,5-dimethylthiazol-2-yl)-2,5-diphenyltetrazolium bromide (MTT) (Sigma-Aldrich) were added to the cells in 96-well plates and incubated for 4 h. The solution in the wells was then replaced with 100 μl dimethyl sulfoxide (DMSO) and incubated for 10 min in a CO_2_ incubator (Thermo Fisher Scientific). The absorbance of the solution in the wells was determined using a microplate reader (iMark BioRad) at a wavelength of 595 nm.

### Differentiation assays

ADSCs at passage 4 (1 × 10^5^) were cultured on silk fibroin scaffold in various medium (StemPro chondrogenesis differentiation kit as positive control, 10% FBS in DMEM as negative control and 10% PRP in DMEM). The cells were analysed on days 1, 3, 5, 7, 14, and 21 for GAG levels and mineralization. The TGF-β level was measured on days 7, 14, and 21, and the surface protein expression compared to the stem cell monolayer before culture on a silk fibroin scaffold (passage 3) was determined on day 21.

#### Secretion of TGF-β1

Each group’s medium of ADSC-SS was removed and the cells were washed three times using HBSS followed by cell starvation in DMEM low glucose medium which was incubated at 37 °C in 5% CO_2_ for 24 h. The medium was then aspirated and used for determination of TGF-β1 levels and the total protein concentration secreted by ADSC-SS using ELISA (R&D system, USA) and Pierce™ Bicinchoninic acid (BCA) Protein Assay Kit (Thermo Scientific, USA), respectively, as aforementioned.

#### Glycosaminoglycans (GAG) analysis

This test was done to measure the GAG in ADSC-SS indicating chondrogenesis using alcian blue stain. Alcian blue is used to stain acidic polysaccharides, such as GAG. The cells were fixed in 4% paraformaldehyde for 15 min. This 4% paraformaldehyde was then removed and 1% alcian blue in 3% acetic acid was added to the fixed cells for 30 min. Alcian blue was then removed and washed thrice with 3% acetic acid for 2 min. The de-ionizing solution was then added and incubated for 2 min. The washed cells were then mixed with 100 μl of 1% sodium dodecyl sulphate (SDS) and mixed in a shaker at 200 rpm for 30 min. The GAG content was determined based on the absorbance value of this solution at a 595 nm wavelength on a microplate reader (iMark Bio-Rad).

#### Mineralization

The purpose of mineralization test is to determine the level of deposited Ca^2+^ in cells. This test for osteogenesis confirms the differentiation of ADSC-SS in each treatment group. The mineralization test was performed as described previously [17] with modifications. At each analysis, ADSC-SS were fixed in 4% paraformaldehyde for 15 min, followed by Alizarin Red S staining and washing thrice with HBSS (Gibco, USA). Next, 10% acetic acid was added to the stained cells. The mineral content was read by absorbance values at 415 nm wavelength using a microplate reader (iMark Bio-Rad).

#### Expression of specific stem cell surface protein markers

This test was performed to determine the consistency of the differentiation direction of ADSC-SS into chondrocytes, which was analysed on day 21. The protocol and analysis of surface marker proteins was performed referring to the BD Stemflow™ hMSC Analysis Kit (BD Biosciences). The expression of cluster of differentiation (CD) 73, CD90, CD105, and CD34/CD45/CD11b/CD19 stem cell surface marker proteins in 1 × 10^5^ monolayer cultured ADSCs at passage 3 (ADSC-MN-P3) was compared to that in ADSCs cultured on a silk fibroin scaffold at passage 4 (ADSC-SS-P4). The sample was incubated with a stem cell surface protein antibody for 30 min and then washed with running buffer. The sample was then centrifuged at 1200 rpm for 5 min. The supernatant was removed, the cell pellet was suspended with 100 μl running buffer, and analysed by flow cytometry (Miltenyi Biotec). The percentage result of surface marker protein expression in three groups from ADSC-SS-P4 was compared to that in ADSC-MN-P3.

### Gene expressions on mRNA level

#### Primer design

The accession numbers of glyceraldehyde-3-phosphate dehydrogenase (GAPDH), type 2 collagen, aggrecan, and type 1 collagen were from genecards.org. The messenger ribonucleic acid (mRNA) sequence of the genes was downloaded from ncbi.nlm.gov.nih. Primer design was performed on sg.idtdna.com/ with primer parameters including primer length of 20–24 bases, melting temperature, %GC content of 40–60%, amplicon size of 75–150 bp, and the secondary structure (hairpin, self-dimer, or repeat). Primer sequences are described in Table 1.

**Table 1:** Primer sequences

Gene		Accession number	Base	Base pairs (bp)
GAPDH	Forward	NM_001256799.2	CAAGAGCACAAGAGGAAGAGAG	22
	Reverse		CTACATGGCAACTGTGAGGAG	22
Type 2 collagen	Forward	NM_001844.4	GAACCCAGAAACAACACAATCC	22
	Reverse		CATTCAGTGCAGAGTCCTAGAG	21
Aggrecan	Forward	NM_001135.3	CAGAATGGGAACCAGCCTATAC	22
	Reverse		GCCTTCTGTACTTTCCTCTGTT	22
Type 1 collagen	Forward	NM_000088.3	AGAGTGGAGCAGTGGTTACTA	21
	Reverse		GATACAGGTTTCGCCAGTAGAG	22

#### Reverse transcription-quantitative polymerase chain reaction (RT-qPCR)

RT-qPCR was performed using a standard protocol from the MIQE (minimum information for publication of quantitative real-time PCR experiments) guide [18]. ADSC-SS in the PRP group and controls (1 × 10^5^) were harvested on days 7, 14, and 21. RNA isolation was performed using SV Total RNA Isolation System (Promega, USA), following the manufacturer’s instructions. Synthesis of complementary deoxyribonucleic acid (cDNA) was performed following the manufacturer’s protocol for the GoTaq® 2-Step RT-qPCR System (Promega, USA). The mRNA expression of GAPDH, type 2 collagen, aggrecan, and type 1 collagen was analysed using SsoAdvanced™ Universal SYBR® Green Supermix (Bio-Rad, USA), with the following cycle conditions: an initial denaturation step of 95 °C for 2 min, followed by 40 cycles of denaturation at 95 °C for 15 s, and annealing at 54.4 °C for 1 min, and a final extension at 60–95 °C for 5 s. The result of qPCR was analysed following the 2^-ΔΔCT^ method described by Livak and Schmittgen [19].

### Localization and abundance of type 2 collagen using immunocytochemistry

Immunocytochemistry protocol was followed as described previously [20] with modification. The three groups of ADSC-SS PRP and control groups on days 7, 14, and 21 were fixed with serial methanol concentrations in DMEM (Gibco, USA) (50%, 70%, 80%, 90%) for 5 min each at 20 °C. ADSC-SS was then fixed with 100% methanol for 20 min at − 20 °C. The fixation was continued with 50% methanol-phosphate-buffered saline (PBS) for 5 min at − 20 °C temperature, followed by washing thrice with HBSS (Gibco, USA) at room temperature. The fixed ADSC-SS was then stabilized with Tween 20 in PBS (PBST, 0,05% Tween concentrate) and blocked with 3% bovine serum albumin (BSA) in PBST. The blocking for ADSC-SS was followed by overnight incubation with the primary antibody against type 2 collagen (Rabbit Anti-Collagen II Antibody (Abcam, UK) (the antibody concentration was 1:200)) in a water bath. The next step was washing the sample thrice using PBS/de-ion. The washed ADSC-SS was then incubated with secondary goat anti-rabbit IgG H&L Alexa Flour 488 antibody (Abcam, UK) (the antibody concentration was 1:200), followed by three washes using PBS/de-ion. The washed ADSC-SS was then incubated in PBS-de-ion containing 4′,6-diamidino-2-phenylindole (DAPI) at 5 μg/ml. Images were acquired and observed randomly using the Olympus confocal microscope type Fv1200 with Fluoview software at 3 points by sectioning at ~ 40 μm of 20 slices for 1 mm thickness. The percentage value from the average of type 2 collagen intensity per three comprehensive areas in the image was determined using ImageJ software (National Institutes of Health, USA).

### Statistical analysis

The data from each parameter was presented as average ± standard deviation (SD) in the tables and charts. Statistical analysis began with normality and homogeneity testing. The differences were analysed (*p* < 0.05) using a parametric statistical test analysis of variance (ANOVA) and it was applied to the proliferation and differentiation test of ADSC-SS, the cell surface marker protein levels in chondrocyte progenitors, measurement of mRNA expression, and the abundance of type 2 collagen.

## Results

### Stromal vascular fraction (SVF)

**Fig. 1:**
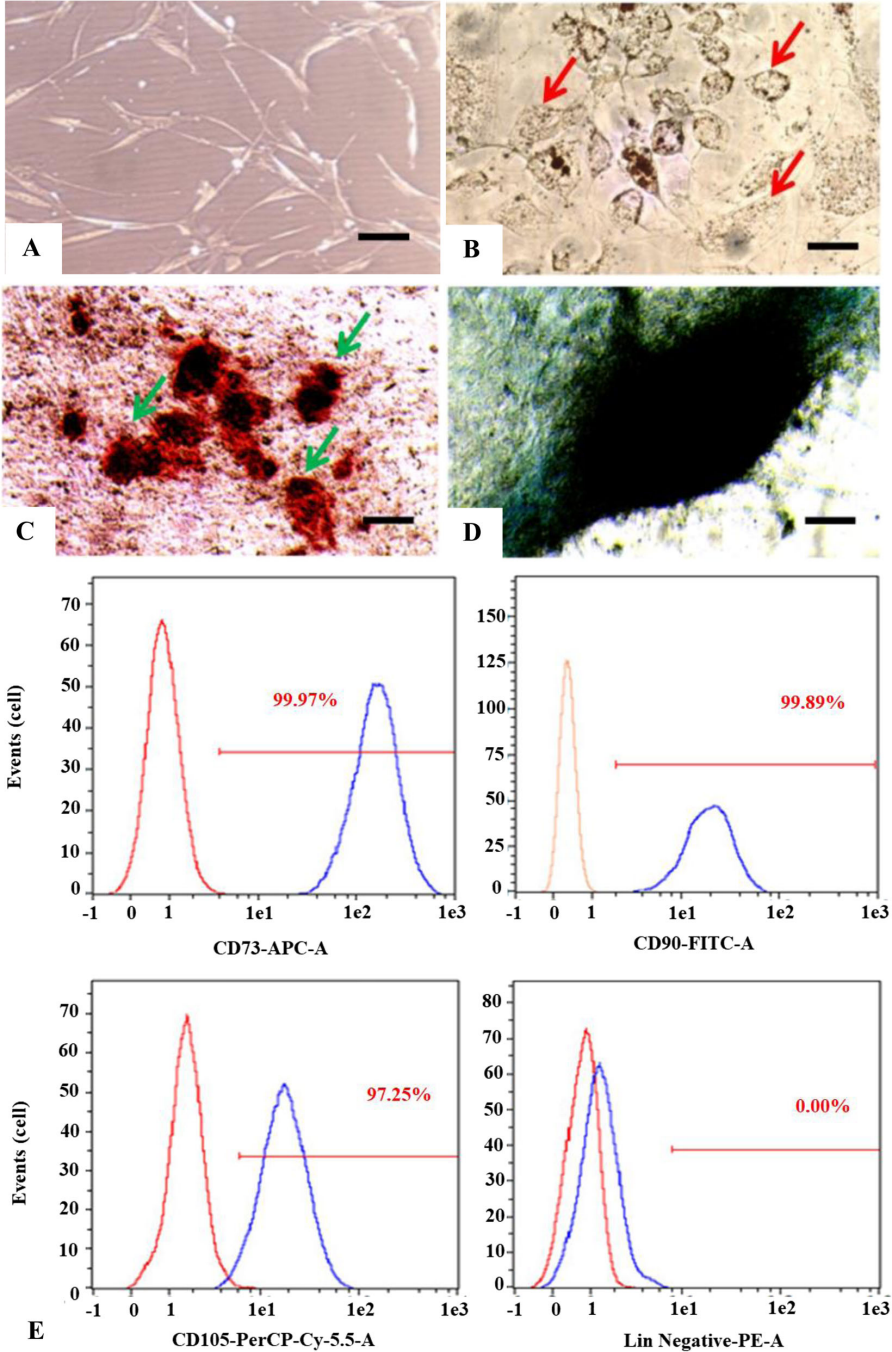
Isolated cells from adipose tissue at passage 1 day 3 showed fibroblast-like morphology (**a**); multipotency of cells at passage 2 isolated from adipose tissue: differentiation into adipocytes characterized by lipid droplets (red arrows) using Oil Red O staining (**b**); osteocytes characterized by calcium deposits (green arrow) using alizarin red staining (**c**); chondrocytes formed micromass-like structures (**d**) using alcian blue staining (black bar = 100 μm) Optilab Microscope, Nikon; Cells at passage 3 expresses CD73 (99.97%), CD90 (99.89%), CD105 (97.25%) and CD45/CD34/CD11b/CD19/HLA-DR less than 2%. Blue peak histogram indicates Fig 2. Proliferation of ADSCs cultured on silk fibroin scaffold with 500 μm pore size (ADSC-SS); *Y* axis is the proliferation rate of ADSC-SS, *X* axis indicates the observation day. The proliferation rate of ADSC-SS on various media is gradually increased. On day 21, the proliferation rate of ADSC-SS PRP group is significantly higher than negative and positive controls, respectively (*p < 0.05 indicates statistical significance). Sample and red peak histogram indicates isotype control (**e**)

The cell number of the SVF including red blood cells in the total count from donors varied with an average of 7.48 × 10^7^ cells/10 mL adipose tissue and the average of cell viability cell was 99.80%. The demographic of lipoaspirate donors are shown in Additional file 1: Table S1. The morphology of cells isolated from adipose tissue in this study was fibroblast-like and attached to the bottom of the plastic plate (Fig. 1a). This is a characteristic of MSCs. However, to confirm and identify the MSCs characteristics of isolated cells from adipose tissue, multipotency and cell surface marker expression was analysed.

### Multipotency and cell-surface marker expression

Cells isolated from adipose tissue were differentiated into adipocytes, chondrocytes, and osteocytes in specific commercial induction medium (Fig. 1b–d). Cells that differentiated into adipocytes showed accumulation of lipid droplets in the cells (Fig. 1b). Calcium deposits in osteogenic cells isolated from adipose tissue were detected through alizarin red staining (Fig. 1c). Chondrogenic differentiation was identified by micromass formation that showed blue colour upon alcian blue staining (Fig. 1d).

We also observed that these cells expressed CD73, CD90, and CD105 proteins on their surface. In Fig. 1e, the *X* axis shows the fluorescent intensity whereas the *Y* axis shows the number of cells. The red and blue peaks of the histogram represent isotypes and stained cells, respectively. The percentage of positive surface protein expression in a population that is quantified by overlaying with the isotype was more than 95% for CD73, CD90, and CD105 and less than 2% for CD34/CD45/CD11b/CD19 (Fig. 1E). These results indicate that cells isolated from adipose tissue meet the criteria for adipose-derived stem cells (ADSCs) according to the International Society for Cellular Therapy (ISCT) and are hereafter referred to as ADSCs [21]. In addition, the minimum information for studies evaluating biologics in orthopaedics (MIBO) checklist for mesenchymal stem cells is shown in Additional file 1: Table S2.

### Platelet number, TGF-β1 level, and total protein of PRP

The platelet number in PRP was 630 × 10^3^/μl, the TGF-β1 level was 22 ng/ml, and the total protein was 4861 μg/ml (Table 2). Proliferation of ADSCs in PRP supplemented medium was investigated in this study. FBS-supplemented and commercially available chondrogenic medium was used for comparison. The MIBO checklist of platelet-rich plasma is shown in Additional file 1: Table S3.

**Table 2:** The platelet number, TGF-β1 protein level, and total protein concentrations of PRP

Measurement	Batch		Average ± SD
	1	2	
Leucocytes (×10^3^/μl)	0.20	0.40	*0.30 ± 0.14*
Erythrocytes (× 10^3^/μl)	0.01	0.03	*0.02 ± 0.14*
Thrombocytes (×10^3^/μl)	275	985	*630 ± 502*
TGF-β1 level (pg/ml)	12,451	32,834	*22,642 ± 14,413*
Protein total (μg/ml)	3980	5742	*4861 ± 1246*

### Growth curve of ADSC-SS

**Fig. 2:**
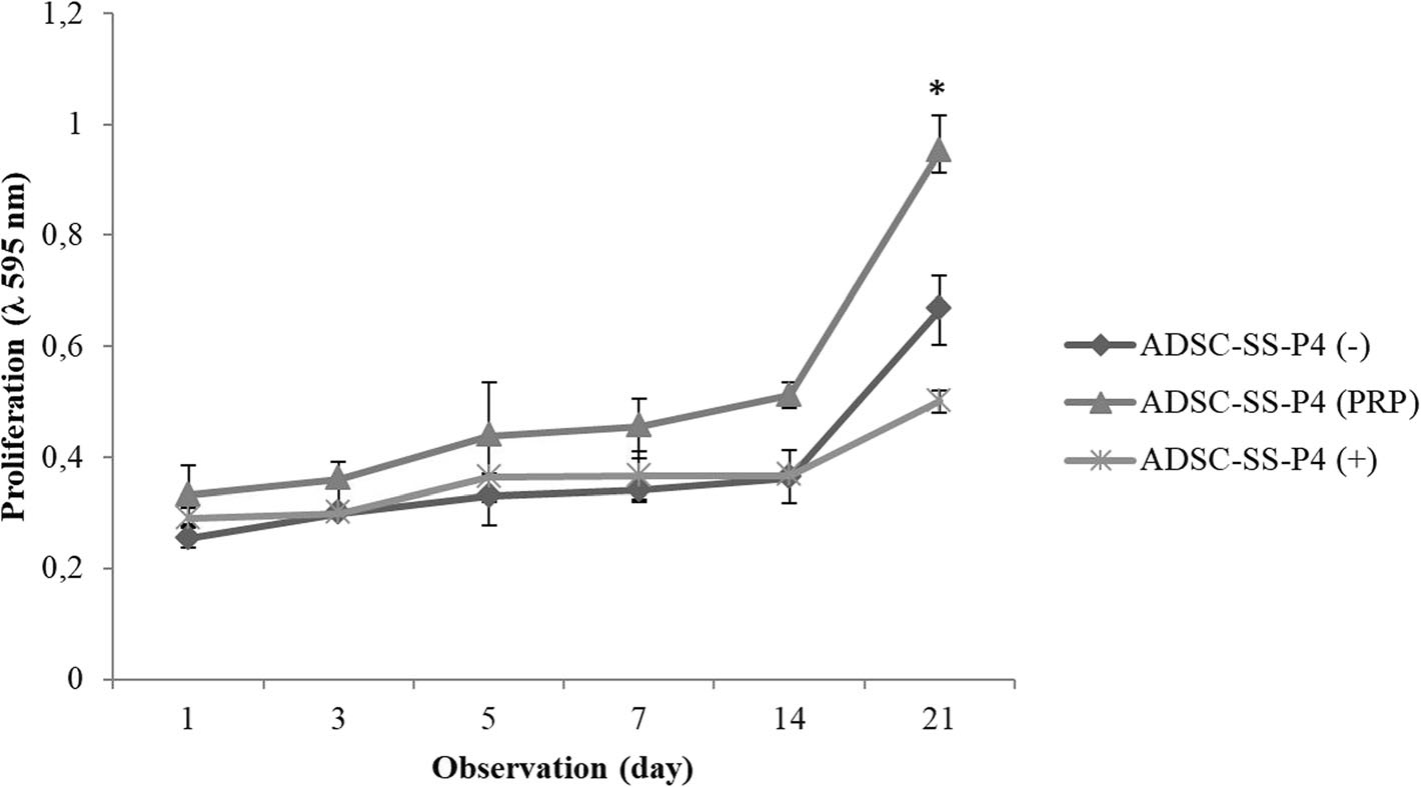
Proliferation of ADSCs cultured on silk fibroin scaffold with 500 μm pore size (ADSC-SS); *Y* axis is the proliferation rate of ADSC-SS, *X* axis indicates the observation day. The proliferation rate of ADSC-SS on various media is gradually increased. On day 21, the proliferation rate of ADSC-SS PRP group is significantly higher than negative and positive controls, respectively (*p < 0.05 indicates statistical significance)

ADSC-SS cultured in various medium for 21 days showed an increase in proliferation from day 1 to day 21. The results showed that the proliferation of ADSC-SS cultured in PRP-supplemented medium showed a higher proliferation rate than those in FBS-supplemented and chondrogenic medium until day 21. The proliferation rate in the ADSC-SS PRP group was significantly higher (*p* < 0.05) compared to the controls on day 21 (Fig. 2). These results indicated that PRP could increase ADSC growth compared to that in FBS-supplemented or commercial chondrogenic medium (StemPro).

### TGF-β level secreted by ADSC-SS

**Fig. 3:**
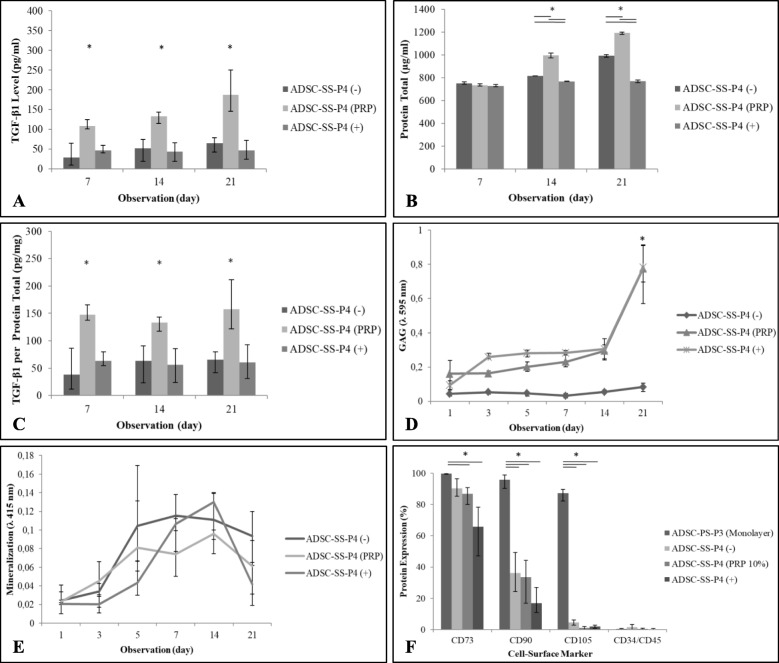
Confirmation of ADSCs differentiation. TGF-β1 level (pg/ml) (**a**), total protein (μg/ml) (**b**), TGF-β1 levels per total protein (pg/mg) (**c**) secreted by ADSC-SS after starvation for 24 h on day 7, 14 and 21 of observation. ADSC-SS in PRP and negative control are gradually increased on day 14 and 21 while ADSC-SS in positive control is stable. The TGF-β1 level which secreted by ADSC-SS PRP group shows significantly different to both of controls on day 7, 14, and 21 of observation; GAG level is dramatically increased in the ADSC-SS PRP and positive control groups and significantly different compared to that in the ADSC-SS negative control on day 21 (**d**); mineralization of ADSC-SS in PRP and control groups are stable in low level for 21 day of observation (**e**); and cell-surface protein expression in ADSC-SS group on day 21 passage 4 compared to that in monolayer ADSCs passage 3, both of ADSC-SS in PRP and positive control are significantly decreased their specific stem cell surface-protein markers (CD73, CD90, CD105) compared to that the ADSC-SS-PS (monolayer) (**f**). (*p < 0.05 indicates statistical significance)

The concentration of TGF-β1 secreted by ADSC-SS during culture was measured at days 7, 14, and 21. To calculate the TGF-β1 secreted by ADSC-SS only apart from TGF-β1 in the PRP, the medium of ADSC-SS was removed and the cells were washed three times using HBSS followed by cells starvation in DMEM low glucose medium which was incubated at 37 °C in 5% CO_2_ for 24 h. The results showed that secretion of TGF-β1 (pg/ml) and total protein (μg/ml) in ADSC-SS was significantly higher in PRP-supplemented medium than in FBS-supplemented and chondrogenic medium (*p* < 0.05) (Fig. 3a, b). We also observed a significant increase in the total protein content after 14 and 21 days of culture in PRP-supplemented and FBS supplemented groups, compared to the chondrogenic medium group (*p* < 0.05) (Fig. 3b). The TGF-β1 level per total protein (pg/mg) in the ADSC-SS PRP group was significantly higher compared to that in the control groups (*p* < 0.05) (Fig. 3c). The results indicate that PRP supplementation induces increased protein secretion by ADSC-SS including TGF-β1, compared to that by FBS and chondrogenic medium.

### GAG level of ADSC-SS

The GAG level of ADSC-SS in PRP and positive control groups was gradually increased whereas the GAG level in the negative control group was relatively stable until 21 days of observation. The level of GAG on day 21 was significantly higher in the PRP and positive control groups compared to that in the negative control (*p* < 0.05) (Fig. 3d). These results indicate that ADSC-SS in the PRP and positive control group were undergoing chondrogenesis.

### Mineralization level of ADSC-SS

Mineralization of ADSC-SS cultured in all tested medium were stable at low concentration. The third group of ADSC-SS showed stability until day 7 followed by an increase on day 14 and a decrease on day 21 (Fig. 3e). The low level of mineralization in this study suggested that cells did not lead to osteogenic differentiation.

### Surface protein marker expression in chondrocyte progenitors

To evaluate the change in mesenchymal stem cell (MSC) surface marker expression upon chondrogenesis, the surface markers on ADSCs were measured on day 21. The results showed alteration in CD73, CD90, and CD105 expression on all ADSC-SS compared to that in monolayer ADSCs cultured on polypropylene. These data indicate that the silk fibroin scaffold supported the cell differentiation.

The results confirmed that passage 3 (P3) ADSCs in a monolayer substrate (ADSC-MN) culture grown in negative control medium expressed higher CD73 levels compared to ADSC-SS P4 cultured in PRP or control medium and significantly higher levels compared to ADSC-SS P4 cultured in PRP and positive control medium (*p* < 0.04). CD90 and CD105 expression on ADSC-MN P3 was significantly higher than that on ADSC-SS P4 cultured in PRP or control medium (*p* < 0.05). The negative marker protein (CD34/CD45/CD11b/CD19) expression of ADSC-SS P4 was stable at low levels (less than 2%) compared to that in ADSC-MN P3 (Fig. 3f).

### mRNA expression of type 2 collagen, aggrecan, and type 1 collagen

The mRNA expression of type 2 collagen in the ADSC-SS PRP group was increased dramatically and was significantly different compared to that in the ADSC-SS negative control on days 7 and 21 (*p* < 0.05). The mRNA expression of type 2 collagen in the ADSC-SS PRP group was higher compared to that in the ADSC-SS positive control group on days 7 and 21 but was significantly lower than that in the positive controls on day 14 (*p* < 0.05). The mRNA expression of aggrecan in the ADSC-SS PRP group was increased on day 14 and decreased on day 21. The gene expression of aggrecan on day 14 was also significantly higher compared to that in the negative control (*p* < 0.05) and was significantly lower compared to that in the positive control (*p* < 0.05).

**Fig. 4:**
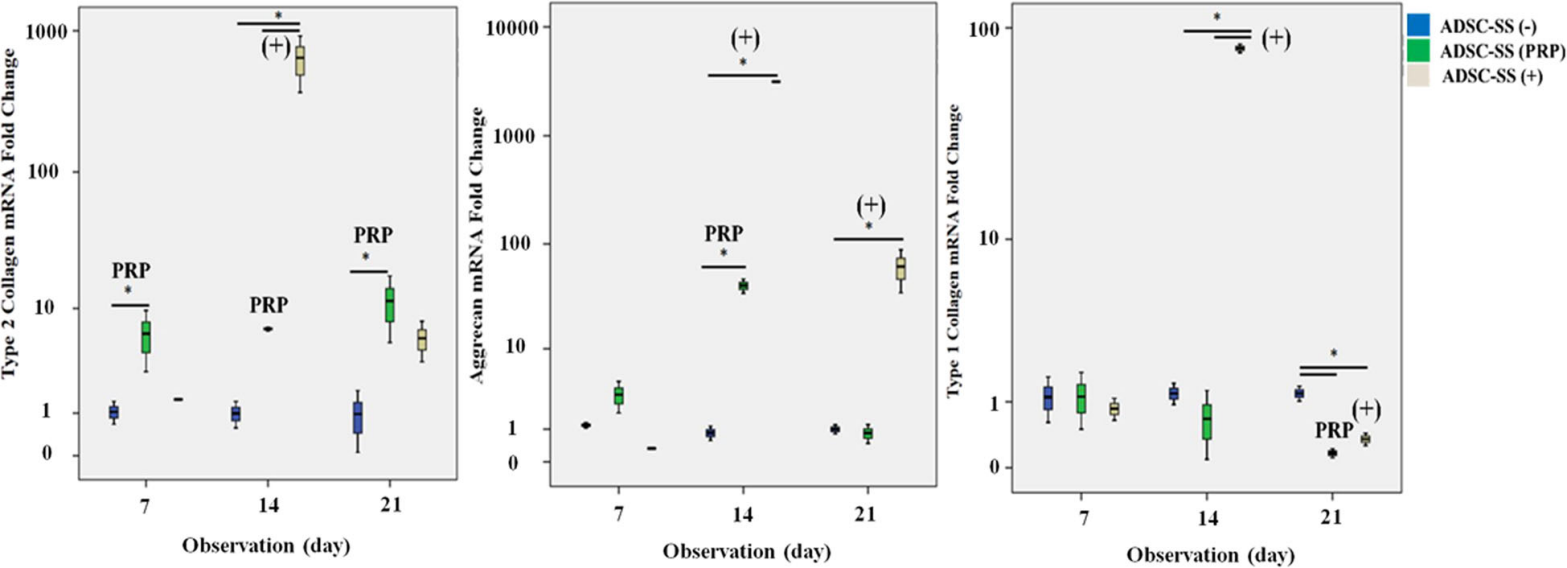
Relative mRNA expression of type 2 collagen (**a**), aggrecan (**b**), and type 1 collagen (**c**) in ADSC-SS PRP and control groups on day 7, 14, and 21. On day 21, ADSC-SS with PRP supplementation induced highest level of type 2 collagen compared to positive and negative controls and expressed low level of aggrecan or type 1 collagen. (*p < 0.05 indicates statistical significance)

The expression of aggrecan in the ADSC-SS positive control group was significantly higher on day 14 compared to that in the PRP and negative control groups (*p* < 0.05) and was significantly higher on day 21 compared to that in the negative control (*p* < 0.05). The mRNA expression of type 1 collagen in the ADSC-SS PRP group was stable until day 14 and was significantly lower compared to the negative control on day 21 (*p* < 0.05). However, the gene expression of type 1 collagen in the ADSC-SS positive control was dramatically increased and significantly higher compared to that in the ADSC-SS PRP and negative control groups on day 14 (*p* < 0.05) and significantly higher compared to the negative control on day 21 (*p* < 0.05) (Fig. 4).

### Immunocytochemistry of type 2 collagen protein

**Fig. 5:**
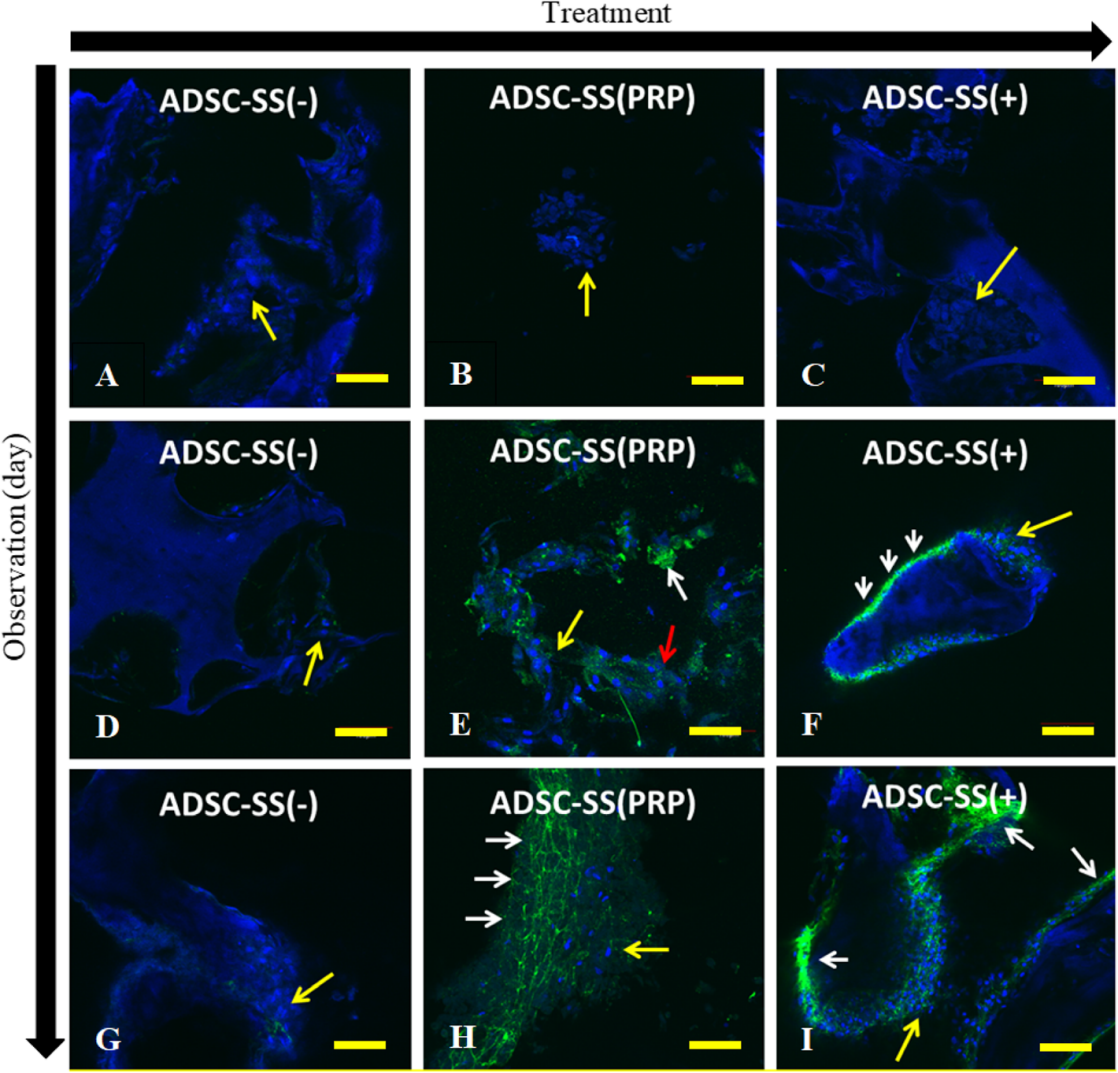
Immunohistochemistry for type 2 collagen (green) in ADSC-SS cultured on medium with 10% FBS (negative control), 10% PRP, and StemPro (positive control) on days 7 (**a**–**c**), 14 (**d**–**f**), and 21 (**g**–**i**); cell nucleus (oval shape) in blue colour (DAPI) is shown by yellow arrows; the silk fibroin scaffold was also stained blue. Type 2 collagen in the cytoplasm is shown by a red arrow; type 2 collagen was outside the cells is shown by white arrows. Cells were observed at × 200 magnification on an Olympus Fv1200 confocal microscope (yellow scale bar = 100 μm)

Cells cultured on silk fibroin scaffold were found within ~ 450 μm depth using optical sectioning, which indicated that the silk fibroin scaffold supported ADSCs proliferation and infiltration into the scaffold. The silk fibroin scaffold also supported chondrogenic differentiation characterized by the presence of type 2 collagen in cells (Fig. 5). In Fig. 5, ADSC-SS in the negative control group showed almost no detectable presence of type 2 collagen whereas ADSC-SS in the positive control group showed the presence of type 2 collagen outside the cells. The presence of type 2 collagen in the ADSC-SS in the PRP and positive control groups increased on days 14 and 21. The presence of type 2 collagen on day 14 in ADSC-SS from the PRP group was observed both inside and outside the cell. On day 21, type 2 collagen in ADSC-SS in the PRP group was highly expressed outside the cells. The presence of type 2 collagen protein in the ADSC-SS positive control group which was observed on days 14 and 21 was outside the cells (Fig. 5).

**Fig. 6:**
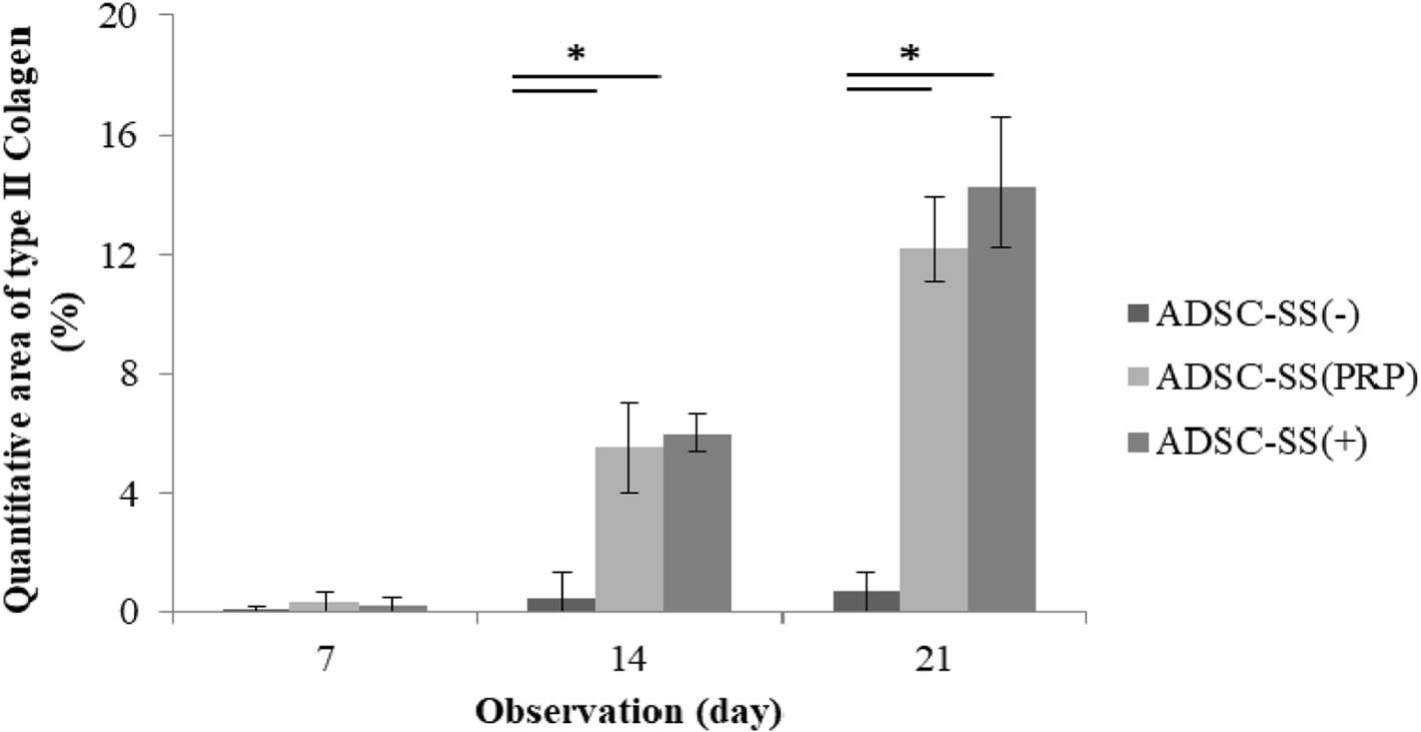
Percentage of type 2 collagen intensity in ADSC-SS PRP and control groups on days 7, 14, and 21. ADSC-SS PRP and positive control expressed significantly higher type 2 collagen percentage on day 14 and 21 compared to negative control group (*p < 0.05 indicates statistical significance)

The percent intensity of type 2 collagen in the treatment groups was very low on day 7. The intensity percentage of type 2 collagen in ADSC-SS with PRP and in the positive control groups was significantly higher compared to that in the negative control group on days 14 and 21. On day 21 (*p* = 0.0003), the intensity percentage of type 2 collagen in ADSC-SS and the positive control groups was 12.26% and 14.27%, respectively (Fig. 6).

Percentage of type 2 collagen protein intensity in ADSC-SS PRP and positive control groups on day 21 was significantly increased compared to that on day 7 and 14 (*p* < 0.04). The presence of an extracellular matrix (ECM) of type 2 collagen in this study indicates the chondrogenesis in the ADSC-SS PRP and positive control groups on days 14 and 21.

**Fig. 7:**
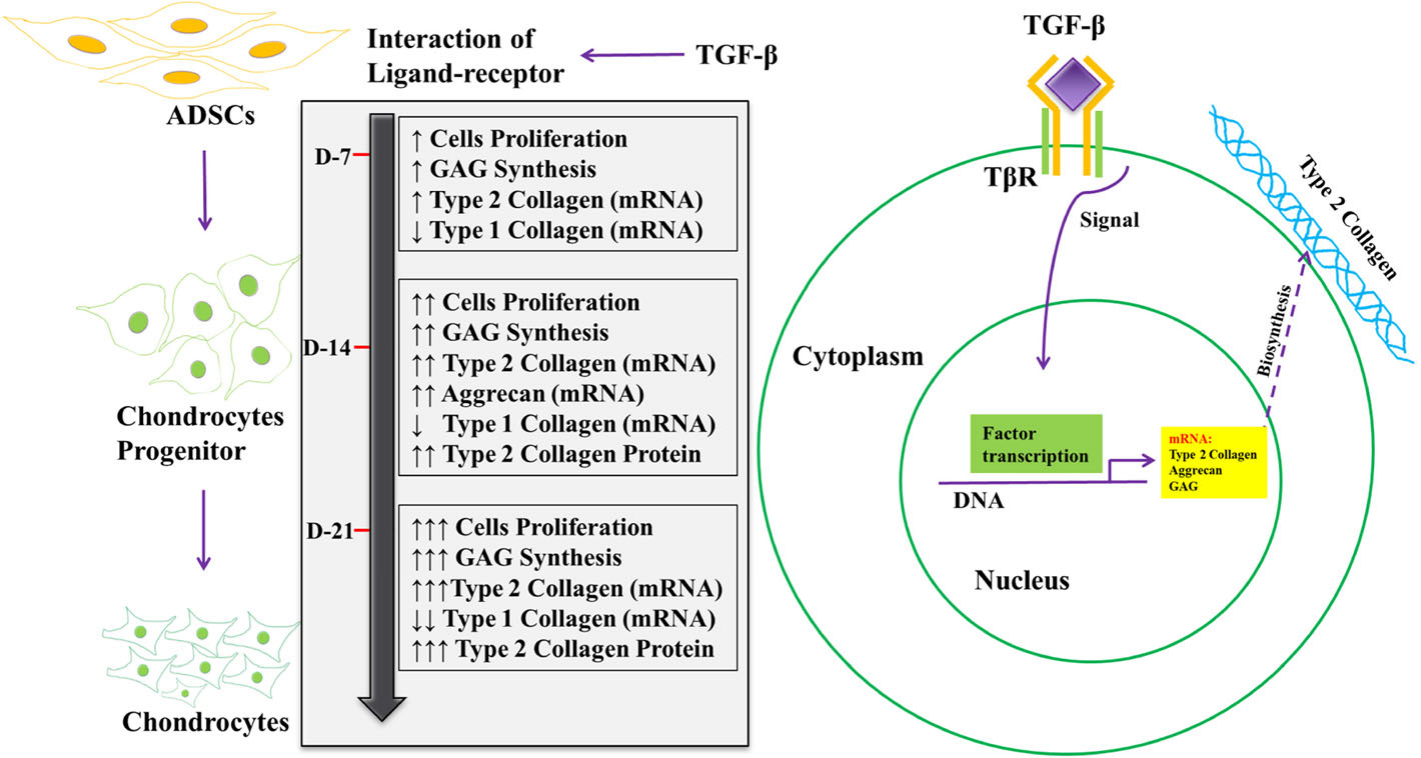
Hypothesis mechanism that occurred in the ADSC-SS PRP group. TGF-β protein in the medium is suggested to be recruited by the ADSC-SS PRP group to induce condrogenesis on days 7, 14, and 21 of observation. Up (↑) and down (↓) arrow in grey box showed the higher (↑) or lower (↓) concentration of mRNA/protein that measured

Based on this study, the mechanism underlying the increased chondrogenesis in the ADSC-SS PRP group was suggested as follows (Fig. 7). TGF-β1 protein is recruited by the cells to induce the expression of chondrogenesis-related proteins. The ADSC-SS PRP group showed an increase in the proliferation rate, GAG levels, and expression of type 2 collagen at the mRNA and protein levels on days 14 and 21, whereas it showed an increase in the expression level of aggrecan at the mRNA level on day 14 and a decrease in its expression level on day 21. Furthermore, the expression of type 1 collagen was detected at a low level on days 7 and 14 and was significantly decreased on day 21.

## Discussion

ADSCs are characterized as fibroblast-like, multipotent (differentiate into several types of cells, like adipocytes, chondrocytes, osteocytes) cells that express CD73, CD90, and CD105 (> 95%) and no CD34, CD45, CD11b, and CD19 (< 2%) [22, 23]. ADSC differentiation into adipocytes are marked by lipid droplets, which are markers of adipogenesis [24]. Furthermore, chondrogenesis and osteogenesis of ADSCs were determined through micromass formation [9] and the accumulation of calcium deposits [25], respectively. These results indicate that cells isolated from fat tissue were ADSCs. Another component that was characterized was PRP. Studies have reported that 1 × 10^9^ platelets/ml in PRP can induce chondrogenesis as well as osteogenesis. The one thing that should be noted for chondrogenesis using PRP is the TGF-β1 level [26–28]. In addition, previously studies reported that the TGF-β1 level which is 10–40 ng/ml in ranges supports chondrogenesis ADSCs [29–31].

In this study, the average of platelet number was below 10^9^ platelet/ml but the TGF-β1 levels of PRP were in the range of the optimal concentration to induce chondrogenesis. Our preliminary study showed that 10% PRP induced chondrogenesis in human ADSCs as determined by alcian blue staining [32]. Furthermore, ADSCs cultured on silk fibroin scaffolds (ADSC-SS) in 10% PRP medium showed highest proliferation rates compared to the controls, suggesting that PRP contains plenty of mitogenic factors. Mitogen factors such as fibroblast growth factor (FGF), platelet-derived growth factor (PDGF), TGF-β, and macrophage inhibitory factor (MIF) play a role in the growth of ADSCs are present in PRP [33–36]. In this study, the TGF-β1 level was measured, but the measurement for other factors was not performed. However, the results of the total protein assay indicate that the total protein level is approximately 5.5 × 10^6^ times higher than the TGF-β1 level.

The high content of mitogen factors in PRP resulted in ADSC aggregation earlier than that in both controls. Cell aggregation is the first stage where cartilage-specific protein markers are produced [37]. ADSC-SS in 10% PRP medium also secreted higher levels of TGF-β1 compared to the controls suggesting that cells were undergoing the chondrogenesis process. Moreover, increasing total protein levels in ADSC-SS PRP and in the negative control group on days 14 and 21 probably resulted due to secretion of other proteins that act as mitogen factors. However, increased TGF-β1 and total protein levels were not found in the ADSC-SS positive control group until day 21 of observation. This might be caused by the fact that the positive control medium contained an optimal level of TGF-β1 for the chondrogenesis process. Therefore, ADSCs were not induced to secrete TGF-β1. Furthermore, the GAG level in the ADSC-SS PRP and positive groups was significantly higher (*p* > 0.05) than that in the negative control group on day 21. The increasing level of GAG is a sign that the chondrogenesis process had occurred [38, 39]. In this study, ADSC-SS in PRP and the positive control did not differentiate into osteocytes as proven by mineralization analysis.

After 21 days, only ADSC-SS in the PRP and positive control groups showed reduced CD73, CD90, and CD105 expression, which might indicate that the cells were in the differentiation process. Reduction in cell-surface markers of MSCs during differentiation into chondrocytes has also been reported in other studies. A similar study inducing chondrogenesis in bone marrow-derived mesenchymal stem cells (BMSCs) cultured on alginate scaffolds showed a significant decrease in CD90 and CD105 [40]. Another study proved that chondrogenesis in MSCs decreased the expression of CD73, CD90, and CD105 [41, 42]. However, the negative control group also showed a significant decrease in CD90 and CD105 expression, which suggested early differentiation of ADSCs into other specific progenitors. We could not predict the differentiation lineage of ADSC-SS in the negative control group because the differentiation was not supported by chondrogenesis or osteogenesis data. Moreover, decreasing CD90 and CD105 was suggested to be caused by the interaction of the cells with the silk fibroin scaffold. We were unable to elucidate the function of the silk fibroin scaffold in differentiation based on this study. Therefore, further studies related to the advantages of using silk fibroin scaffold for cell differentiation of ADSCs are needed. Gene expression at the mRNA level was assessed to confirm the differentiation process.

The mRNA expression of chondrogenesis marker is induced by several effector proteins initiated by transcription factor genes [43]. In this study, type 2 collagen and aggrecan were used as chondrocyte markers whereas type 1 collagen was an osteocyte marker. Increasing gene expression of type 2 collagen and aggrecan at mRNA level in the ADSC-SS PRP and positive control groups suggested that chondrogenesis had occurred. In this study, type 2 collagen and aggrecan were synthesized by cells through recruitment of TGF-β protein in the medium. Gene expression of type 1 collagen showed the lowest level for the PRP group on every observation day whereas type 1 collagen expression in the positive control group was increased on day 14 followed by a significant decrease on day 21, suggesting that chondrogenesis suppressed the synthesis of the osteogenesis marker protein. The chondrocyte ECM composed of type 2 collagen and aggrecan maintain chondrogenesis by suppressing the expression of osteogenesis marker proteins [44, 45].

The proliferation, infiltration, and differentiation of ADSCs were also confirmed by immunocytochemistry. Infiltration of ADSCs cultured on silk fibroin/hydroxyapatite scaffolds with ~ 112 μm pore size showed increased growth and ECM secretion of chondrocytes [46]. Other studies using various types of cells and scaffolds including human MSCs, 3 T3 fibroblasts, and MC3T3-E1 cells in poly ε-caprolactone (PCL)/gelatin scaffolds, porous poly (L-lactide) (PLLA), and PLLA/polyethylene oxide (PEO) showed that cells could infiltrate ~ 300, ~ 350, and ~ 600 μm size of pores [47–49]. The results in this study showed that the ADSC-SS PRP and positive control were proliferated, infiltrated, and differentiated into chondrocytes supported by the presence of type 2 collagen. The presence of type 2 collagen in the chondrogenesis of BMSCs cultured on silk fibroin is also reported [50]. Chondrogenesis of BMSCs in poly-lactide-co-glycolide (PLGA) scaffolds showed approximately ~ 35% of type 2 collagen for the culture duration of 42 days [20]. However, in this study, the percentage of type 2 collagen was up to ~ 12.26% in 21 days culture.

**Fig. 8:**
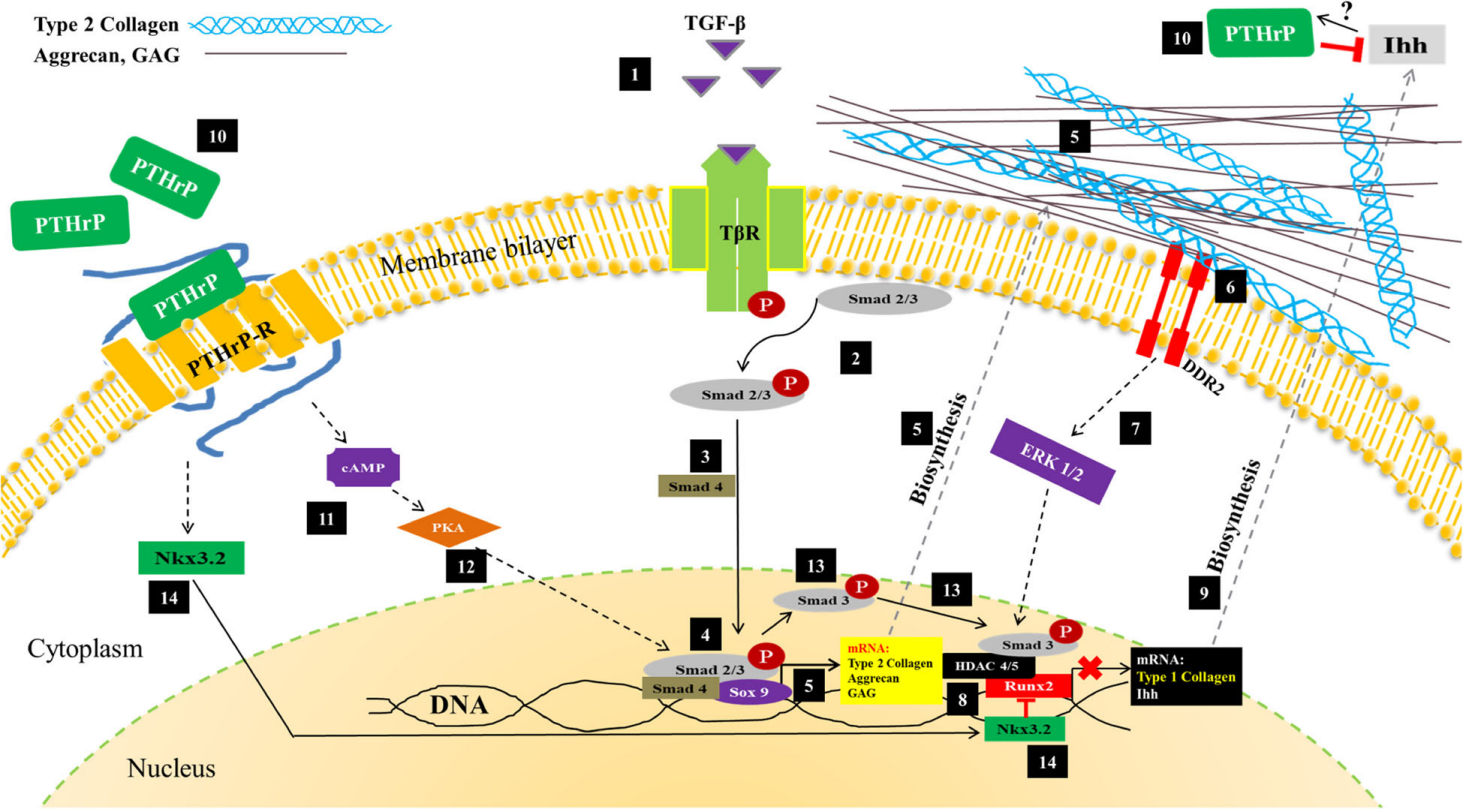
Diagram hypothesis mechanism of ADSC-SS condrogenesis in PRP and positive control groups (1–14). TβR TGF-β receptor, DDR2 discoidin receptor 2 domain, ERK extracellular-signal-regulated kinase, Runx2 runt-related transcription factor 2, PTHrP parathyroid hormone-related protein, Sox Sry-related HMG box, HDAC histone deacetylation, Nkx3.2 NK3 homeobox 2, cAMP cyclic adenosine monophosphate, PKA/C protein kinase A/C

When the results of this study are compared to similar studies, the hypothesized mechanism is suggested as follows (Fig. 8): TGF-β molecules in the medium of PRP and the positive control as well as those secreted by the cells interact with the TGF-β receptor. The TGF-β ligand-receptor interaction initiates phosphorylation of the Smad 2/3 effector protein. Smad 2/3 protein will make a complex with Smad 4 and then translocate into the nucleus to express genes regulated by the sry-related HMG box (Sox 9) transcription factor. Sox 9 protein will induce the gene expression of type 2 collagen, aggrecan, and GAG. These genes will then be translated and secreted into the protein ECM of cartilage [15].

In this study, the transcription and translation process of type 2 collagen in the ADSC-SS PRP group suggested that they did not occur at the same time. Type 2 collagen in the ADSC-SS PRP group was only found in the cytoplasm and outside the cells on day 14. This suggested a mechanism of delayed protein synthesis [51]. In the final stage of chondrogenesis, GAG and type 2 collagen form the ECM of chondrocytes. Once the chondrocyte is mature, type 2 collagen interacts with the discoidin receptor 2 domain (DDR2) receptor. This interaction activates extracellular-signal-regulated kinase 1/2 (ERK 1/2) and initiates the activation of runt-related transcription factor 2 (Runx2) transcription factor to regulate the expression of genes related to osteogenesis. The osteogenesis genes include type 1 collagen and Indian hedgehog (Ihh) [43].

It is suggested that Ihh protein will activate the synthesis of the parathyroid hormone-related protein (PTHrP) protein, but the mechanism underlying this is unclear. PTHrP protein synthesized by the cells will inhibit Ihh indirectly [43, 52]. Secreted PTHrP protein will interact with the PTHrP receptor, followed by the activation of the cyclic adenosine monophosphate (cAMP) and NK3 homeobox 2 (Nkx3.2) pathways. cAMP will activate protein kinase A (PKA), which will then make a complex with Smad3 protein to de-acetylate Runx2-mediated osteogenesis-related genes. Therefore, the expression of genes related to osteogenesis, such as type 1 collagen, will be suppressed [44, 45, 53, 54]. This mechanism was suggested to explain the increase in type 1 collagen on day 14 followed by the significant decrease in type 1 collagen on day 21 in the ADSC-SS positive control.

## Conclusions

Adipose-derived stem cells cultured on silk fibroin scaffolds with a 500 μm pore size (ADSC-SS) in a medium containing 10% platelet-rich plasma (PRP) were differentiated into chondrocytes, which are characterized by TGF-β1 secretion, and increased the expression of glycosaminoglycan as well as type 2 collagen at the mRNA and protein level.

## Supplementary information

**Additional file 1:** **Table S1.** Demographics of lipoaspirate donors. **Table S2.** Minimum Information for Studies Evaluating Biologics in Orthopaedics (MIBO) checklist for mesenchymal stem cells research. The MIBO Checklist template followed by [55]. **Table S3.** MIBO checklist for platelet-rich plasma research. The MIBO Checklist template followed by [55, 56].

## Data Availability

All relevant data are within the paper.

## Abbreviations

3D: 3 dimension
ADSC-MN: ADSCs in a monolayer substrate
ADSCs: Adipose-derived stem cells
ADSC-SS: ADSCs cultured on silk fibroin scaffolds
ANOVA: Analysis of variance
APC: Allophycocyanin
BCA: Bicinchoninic acid
BMSCs: Bone marrow-derived mesenchymal stem cells
BSA: Bovine serum albumin
cAMP: Cyclic adenosine monophosphate
CD: Cluster of differentiation
cDNA: Complementary deoxyribonucleic acid
CS: Chondroitin sulphates
DAPI: 4′,6-diamidino-2-phenylindole
DDR2: Discoidin domain receptor 2
DMEM: Dulbecco’s modified Eagle’s medium
DMSO: Dimethyl sulfoxide
DS: Dermatan sulphates
ECM: Extracellular matrix
ELISA: Enzyme-linked immunosorbent assay
ERK: Extracellular-signal-regulated kinase
FBS: Foetal bovine serum
FGF: Fibroblast growth factor
FITC: Fluorescein isothiocyanate
GAG: Glycosaminoglycans
GAPDH: Glyceraldehyde 3-phosphate dehydrogenase
HBSS: Hank’s balanced salt solution
HDAC: Histone deacetylase
HLA: Human leukocyte antigen
HS: Heparan sulphates
IFRC: Indonesian red cross society
Ihh: Indian hedgehog
ISCT: International Society for Cellular Therapy
KS: Keratan sulphates
MIF: Macrophage inhibitory factor
MIQE: Minimum information for publication of quantitative real-time PCR experiments
mRNA: Messenger ribonucleic acid
MSCs: Mesenchymal stem cells
MTT: 3-(4,5-Dimethylthiazol-2-yl)-2,5-diphenyltetrazolium bromide
Nkx3.2: NK3 homeobox 2
PBS: Phosphate-buffered saline
PBST: Tween 20 in PBS
PCL: Poly ε-caprolactone
PDGF: Platelet-derived growth factor
PEO: Porous poly (L-lactide)/polyethylene oxide
PerCP: Peridinin-chlorophyll-protein
PKA/C: Protein kinase A/C
PLGA: Poly-lactide-co-glycolide
PLLA: Porous poly (L-lactide)
PRP: Platelet-rich plasma
PTHrP: Parathyroid hormone-related protein
RT-qPCR: Reverse transcriptase-quantitative polymerase chain reaction
Runx2: Runt-related transcription factor 2
SD: Standard deviation
SDS: Sodium dodecyl sulphate
Sox9: Sry-related HMG box 9
SVF: Stromal vascular fraction
TGF-β: Transforming growth factor-beta
