# Supplementary material for: In vitro study of cartilage tissue engineering using human adipose-derived stem cells induced by platelet-rich plasma and cultured on silk fibroin scaffold
Source: Stem Cell Res Ther. 2019 Dec 4;10:369. doi: 10.1186/s13287-019-1443-2 (PMC6894137; doi:10.1186/s13287-019-1443-2)
Supplement: Supplementary file 1 — Additional file 1: Table S1. Demographics of lipoaspirate donors. Table S2. Minimum Information for Studies Evaluating Biologics in Orthopaedics (MIBO) checklist for mesenchymal stem cells research. The MIBO Checklist template followed by [55]. Table S3. MIBO checklist for platelet-rich plasma research. The MIBO Checklist template followed by [55, 56]. [file 13287_2019_1443_MOESM1_ESM.pdf]

Table S1 – Demographics of lipoaspirate donors

Sample	Age	Sex	Anatomical Source (Fat Tissue)	Equipment	Time Between Harvest & Processing	Cell SVF Number per 10 ml Fat Tissue (Cell)	Viability (%)
Donor-1	49	F	Abdominal Subcutaneous	Refrigerate Centrifuge 1 SL40R, Shaker Incubator Unimax1010 Hideolph, Light Microscope CX23 Olympus	Less than 1 hour	81.000.000	97,61
Donor-2	51	F	Abdominal Subcutaneous		Less than 1 hour	108.000.000	98,79
Donor-3	41	F	Abdominal Subcutaneous		Less than 1 hour	45.000.000	99,19
Donor-4	54	M	Abdominal Subcutaneous		Less than 1 hour	65.000.000	99,59
**Average ± SD**	**49 ± 5.56**					**74,750,000 ± 26,612,967**	**98.80 ± 0.85**

Table S2 – Minimum Information for Studies Evaluating Biologics in Orthopaedics (MIBO) checklist for mesenchymal stem cells research. The MIBO Checklist template followed by [55]

Section / Topic	Item	Checklist item	Reported on Page No.
Study Design	1	Study conducted in accordance with CONSORT (RCT), STROBE (cohort, case-control or cross-sectional) or PRISMA (meta-analysis) guidelines	-
	2	Relevant institutional and ethical approval	20
Recipient details	3	Recipient demographics (including age, gender)	Table S1
	4	Comorbidities (including underlying diabetes, inflammatory conditions, pre-existing joint pathology and smoking status)	-
	5	Current anti-inflammatory medications	-
Injury details	6	Diagnosis (including relevant grading system and chronicity)	-
	7	Previous treatments for current injury	-
Intervention	8	Surgical intervention described sufficiently to enable replication	-
	9	Operative findings	-
Donors	10	Donor age	Table S1
Tissue harvest	11	Tissue harvest described sufficiently to enable replication (including anatomical source, equipment, reagents, storage media and environment)	Table S1
	12	Time between tissue harvest and processing	Table S1
Processing	13	Description of tissue processing that makes replication of the experiment possible (including digestion solution concentrations and volumes, duration, agitation and temperature of digestion phase, name of commercial system)	3
	14	If performed, purification described sufficiently to enable replication (including combination and concentration antibodies, equipment, method of confirming purity)	-
	15	Yield with respect to volume of tissue processed	Table S1
Cell Culture	16	If performed, cell culture described sufficiently to enable replication (including conditions, number of freeze-thaw cycles)	3
	17	If performed, predifferentiation described sufficiently to enable replication	3,4
MSC characteristics	18	MSC preparation and source described in title and abstract (e.g. BMMSC, ADSC)	1,3
	19	Cellular composition / heterogeneity	-
	20	Immunophenotype and details of in vitro differentiation tested on batch	7
	21	Passage and percentage viability	7, Table S1
Delivery	22	MSC delivery described sufficiently to enable replication (including point of delivery, volume of suspension and media used as vehicle)	-
	23	If performed, details of codelivered growth factors, scaffolds or carriers	2-3
Post-operative care	24	Rehabilitation protocol sufficiently described to enable replication (including immobilisation and physical therapy)	-
Outcome	25	Outcome assessments include functional outcomes and recording of complications (including infection and tumour). If performed radiographic outcomes, physical examination findings, return to activities and satisfaction	-

Table S3 - MIBO checklist for platelet-rich plasma research. The MIBO Checklist template followed by [55,56]

**Section / Topic**	**Item number**	Checklist item	Reported on Page No
Study design	1	Study conducted in accordance with CONSORT (RCT), STROBE (cohort, case-control or cross-sectional) or PRISMA (meta-analysis) guidelines	-
	2	Relevant institutional and ethical approval	20
Recipient details	3	Recipient demographics (including age and gender)	-
	4	Comorbidities (including underlying diabetes, blood dyscrasia, inflammatory conditions, pre-existing joint pathology and smoking status)	-
	5	Current anti-inflammatory or anti-platelet medications	-
Injury details	6	Diagnosis (including relevant grading system and chronicity)	-
	7	Results of any pre-operative imaging	-
	8	Previous surgical or biological treatments for current injury	-
Intervention	9	Intervention described sufficiently to enable replication	-
	10	Operative findings	-
Whole blood processing	11	Whole blood storage environment (including concentration and volume of anticoagulant, temperature and light exposure)	-
Whole blood characteristics	12	Whole blood platelet, differential leukocyte and red cell analysis of all samples	-
PRP processing	13	PRP processing described sufficiently to enable replication (including commercial kit details and Spin protocol)	-
	14	Platelet recovery rate of protocol	4,8
	15	PRP storage temperature and light exposure	4
	16	Time between blood drawing, PRP processing, activation and delivery	4
PRP characteristics	17	PRP format (for example liquid, gel, membrane)	4
	18	PRP platelet, differential leukocyte and red cell analysis of all samples	4,8
Activation	19	Activation described sufficiently to enable replication (including volume and concentration of activating agent)	-
Delivery	20	Point of delivery (intraoperative and/or postoperative or serial)	-
	21	PRP delivery described sufficiently to enable replication (including volume delivered, concomitant use of stem cells or cytokines, and details of carrier or scaffold)	4,5
Post-operative care	22	Rehabilitation protocol sufficiently described to enable replication (including immobilization and physical therapy)	-
Outcome	23	Outcome assessments include functional outcomes and recording of complications (including infection and need for further surgery). If performed radiographic outcomes, physical examination findings, return to activities and satisfaction.	-

Due to the PRP obtained from Indonesian Red Cross Society, the data about demographics, PRP processing, comorbidities, diagnosis, and whole blood analysis were not allowed to be reported.
